# Evaluation of the Impact of Universal Mupirocin Decolonization in Intensive Care Units on the Utility of Methicillin-Resistant *Staphylococcus aureus* Polymerase Chain Reaction Nasal Swab Testing

**DOI:** 10.1093/cid/ciag183

**Published:** 2026-03-16

**Authors:** Andrea S Ito, Kaitlyn C Reasoner, Sharon K Ong’uti, Edward T Qian, Zhiguo Zhao, Rebecca A Stern, Jeffrey A Freiberg

**Affiliations:** Division of Infectious Diseases, Department of Medicine, Vanderbilt University Medical Center, Nashville, Tennessee, USA; Division of Infectious Diseases, Department of Medicine, Vanderbilt University Medical Center, Nashville, Tennessee, USA; Division of Infectious Diseases, Department of Medicine, Vanderbilt University Medical Center, Nashville, Tennessee, USA; Division of Allergy, Pulmonary, and Critical Care Medicine, Department of Medicine, Vanderbilt University Medical Center, Nashville, Tennessee, USA; Department of Anesthesiology, Vanderbilt University Medical Center, Nashville, Tennessee, USA; Department of Biomedical Informatics, Vanderbilt University Medical Center, Nashville, Tennessee, USA; Department of Biostatistics, Vanderbilt University Medical Center, Nashville, Tennessee, USA; Division of Infectious Diseases, Department of Medicine, Vanderbilt University Medical Center, Nashville, Tennessee, USA; Division of Infectious Diseases, Department of Medicine, Vanderbilt University Medical Center, Nashville, Tennessee, USA; Vanderbilt Institute for Infection, Immunology, and Inflammation, Vanderbilt University Medical Center, Nashville, Tennessee, USA; Department of Pathology, Microbiology, and Immunology, Vanderbilt University Medical Center, Nashville, Tennessee, USA; United States Department of Veterans Affairs Medical Center, Nashville, Tennessee, USA

**Keywords:** MRSA nares, mupirocin decolonization, antimicrobial stewardship, infection prevention, *Staphylococcus aureus*

## Abstract

**Background:**

The reliability of methicillin-resistant *Staphylococcus aureus* (MRSA) polymerase chain reaction (PCR) nasal swab testing in the context of mupirocin nasal decolonization has been questioned. This study investigated whether the negative predictive value (NPV) of MRSA PCR nasal swab testing following mupirocin decolonization was noninferior to testing prior to decolonization.

**Methods:**

This retrospective cohort study included adult inpatients admitted to intensive care units (ICUs) from February 2023 to August 2024 who underwent both MRSA PCR nasal swab testing and universal mupirocin decolonization. Patients were divided into 3 groups based on timing of PCR testing: prior to receiving any doses of topical mupirocin, no more than 7 days after, or >7 days after a first dose. The primary outcome was the NPV of MRSA PCR nasal swab testing.

**Results:**

A total of 1034 patients were included in the primary analysis; 508 (49.1%) had a MRSA PCR nasal swab collected prior to the start of decolonization and 388 (73.8%) and 138 (26.2%) received nasal swabs > 0 to ≤7 days and >7 days after the start of mupirocin decolonization, respectively. The differences between the NPV of MRSA PCR testing in patients with swabs collected prior versus in patients with swabs collected >0 to ≤7 days and >7 days after the start of mupirocin were 0.3% and –0.4% (1-sided 95% CI −.9% and CI −2.3%), respectively.

**Conclusions:**

The NPV of MRSA PCR nasal swabs is not diminished after mupirocin decolonization. MRSA PCR testing within 7 days of initiation of decolonization is noninferior to testing prior to decolonization.

## Teaser

MRSA PCR nasal swab testing remains a reliable tool, with a high NPV across multiple culture locations, in patients who have received universal mupirocin decolonization in an intensive care unit (ICU) in the past 7 days.

**(See the Editorial Commentary by Pérez-Granda and Bouza on pages e249–50.)**

Methicillin-resistant *Staphylococcus aureus* (MRSA) is a serious pathogen, with over 200 000 related adult hospitalizations in the United States in 2021 [1]. Between 1990 and 2021, MRSA was the pathogen responsible for the greatest rise in global deaths associated with and attributable to antimicrobial resistance, and was responsible for 130 000 deaths in 2021 [2]. In patients with suspected hospital-associated or ventilator-associated pneumonia, anti-MRSA coverage is recommended for patients with risk factors for antimicrobial resistance, or in ICUs where methicillin resistant is more common among *S. aureus* isolates [3]. Although MRSA is a rare cause of community acquired pneumonia (CAP) accounting for <5% of cases, up to 38% of patients hospitalized with CAP may also receive anti-MRSA antibiotics [4–8]. Empiric use of the anti-MRSA agent vancomycin has been associated with drug reactions and kidney injury, including nephrotoxicity specifically in patients hospitalized with pneumonia [8–10]. Vancomycin also accrues significant cost related to intensive monitoring and dosing supervision by pharmacy [11]. Reduction of inappropriate vancomycin use provides an opportunity to reduce potential toxicities to patients while also promoting cost savings to the healthcare system.

MRSA colonization of the nares is associated with an increased risk of MRSA in a variety of different infections as the anterior nares act as a *S. aureus* reservoir [12–17]. The lack of MRSA colonization of the nasal cavity has a high negative predictive value (NPV) for MRSA infections in multiple sites, and a growing body of evidence supports using MRSA PCR nasal swabs to guide de-escalation of vancomycin in suspected or proven infections [18–27]. This data is particularly robust for pneumonia, where the NPV of MRSA polymerase chain reaction (PCR) nasal swab testing is 96.5%, with an even higher NPV of 98.1% for CAP, hence the inclusion of a MRSA PCR nasal screening recommendation in the American Thoracic Society and Infectious Diseases Society of America joint CAP guidelines from 2019 [28].

Given the morbidity and mortality associated with MRSA infections, various strategies have been implemented to prevent transmission and reduce MRSA infections. Universal decolonization with mupirocin or povidone iodine in combination with chlorohexidine gluconate is the recommended strategy for all patients in the ICU to reduce the risk of *S. aureus* bloodstream infections [29–32]. However, concerns have been raised over diminished reliability or NPV of MRSA PCR nasal swabs in the setting of concurrent or prior nasal mupirocin decolonization [33].

This concern regarding the potential impact of mupirocin decolonization is significant as it has prompted cessation in the use of MRSA PCR nasal swabs as an antibiotic de-escalation strategy in some cases [34]. However, given the ability of MRSA PCR testing to detect bacterial DNA, even after bacteria are killed, there is presumably a window of time after mupirocin decolonization during which MRSA PCR testing is likely to still be a viable antimicrobial stewardship strategy. To better define this window, a retrospective study was designed to specifically examine the relationship between mupirocin timing and the NPV of MRSA PCR swab testing. Given that the high NPV value of MRSA PCR nasal swab testing has been emphasized as the driving force behind its usefulness, we set out to test the hypothesis that MRSA PCR nasal swab testing in the week following the start of mupirocin decolonization was noninferior to PCR testing prior to mupirocin decolonization with regards to any differences in the NPV.

## METHODS

### Study Design

**Figure 1.**
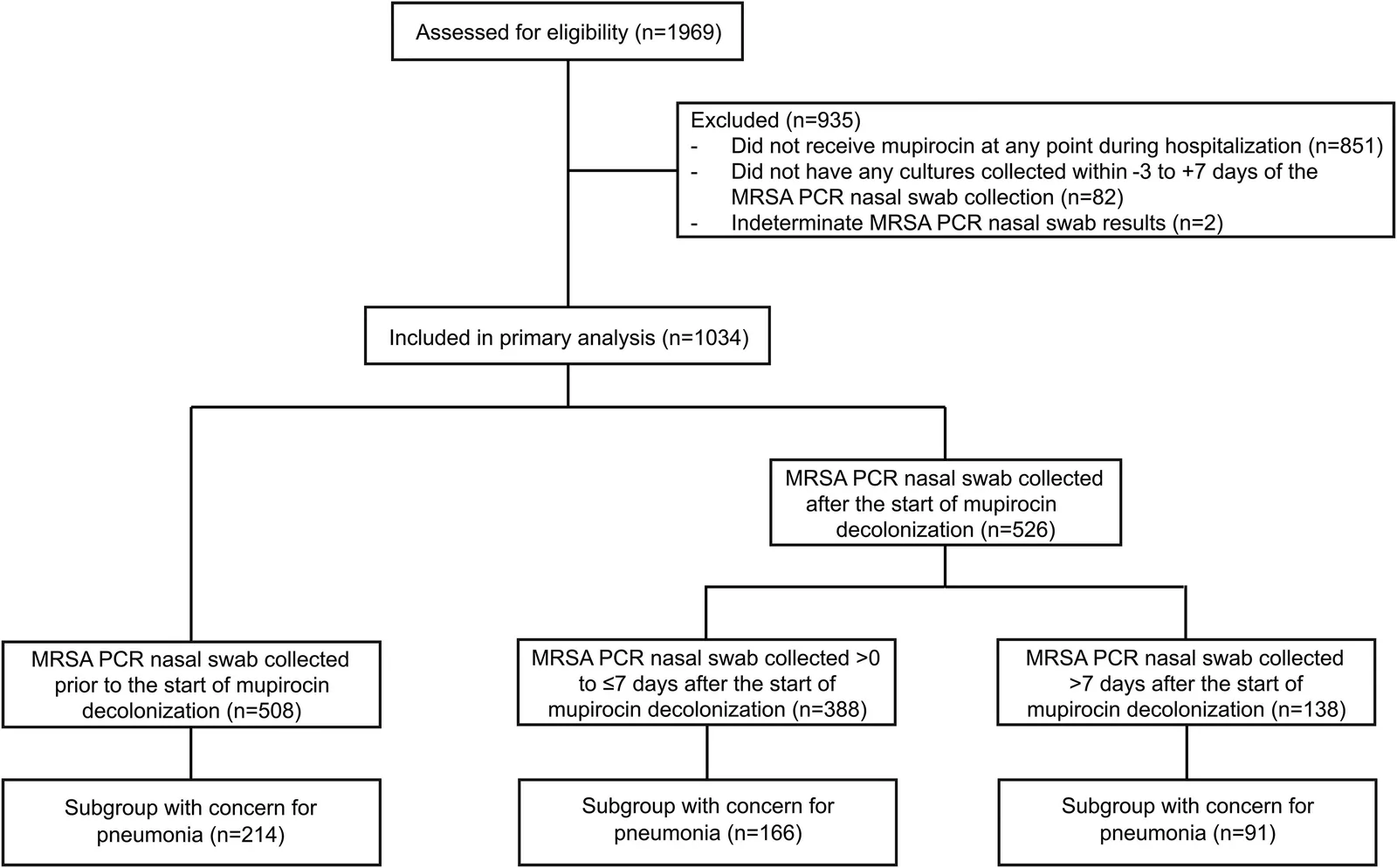
Flow diagram of patients included in the study. Abbreviations: MRSA, methicillin-resistant *Staphylococcus aureus*; PCR, polymerase chain reaction.

We conducted a retrospective cohort study of adult patients admitted to 8 ICUs across 4 hospitals in Tennessee who underwent both MRSA PCR nasal swab testing and mupirocin decolonization during the same hospital admission between February 2023 and August 2024. An initial query identified all hospital encounters in which a patient had a MRSA PCR nasal swab result and was admitted to an ICU during the same admission. Patients who did not receive at least 1 dose of topical mupirocin, did not have an eligible culture collected (defined as any bacterial culture collected up to 3 days before or 7 days after MRSA PCR nasal swab testing), or had an indeterminate result on their MRSA PCR nasal swab were excluded from the primary analysis (Figure 1). This study was approved by the Vanderbilt University Medical Center institutional review board (IRB #241111).

### Data Collection

Patient data including demographics, hospital and ICU length of stay, timing of mechanical ventilation, MRSA PCR nasal swab testing, and mupirocin administration were all extracted from the EHR. Bacterial culture and MRSA PCR nasal swab testing results were electronically extracted, with subsequent manual chart review performed for generic culture orders with an unspecified source. Patients were determined to have reasonable suspicion for pneumonia if, in addition to having a respiratory culture collected, they had documented radiographic findings consistent with pneumonia (including mention of infiltrate, opacities, consolidations, concern for pneumonia, or inability to exclude pneumonia), and they had clinical concern for pneumonia. Clinical concern for pneumonia was considered to be the presence of at least 2 of the following: worsening cough, increased sputum production compared with baseline, subjective dyspnea, respiratory rate >20, temperature <36°C or >38°C, oxygen saturation <90% or increased oxygen requirement over baseline, or a white blood cell count <4000 or >10 000 cells/µL [28].

### Statistical Analysis

Patient characteristics were described as categorical variables using frequencies and percentages or as continuous variables using median and interquartile range. For the purposes of the primary outcome, patients were divided into 3 groups; patients in whom a MRSA PCR nasal swab test was collected prior to receiving any doses of topical mupirocin (Prior group), patients in whom a MRSA PCR nasal swab test was collected after receiving a dose of topical mupirocin, but no more than 7 days after (0–7 day group), and patients in which a MRSA PCR nasal swab test was collected >7 days after a first dose of topical mupirocin (>7 day group). Differences in patient characteristics between the 3 groups were tested using either a Kruskal–Wallis test for continuous variables or a χ^2^ test for categorical variables. Sensitivity, specificity, positive predictive value, and NPVs were reported for all 3 groups. The corresponding 95% confidence intervals were estimated using the hybrid Wilson/Brown method. The size of this cohort enabled a precise evaluation of MRSA PCR testing performance. Specifically, the half-widths of the 95% confidence intervals for the NPV estimates were 1.1%, 1.2%, and 2.6%, corresponding to the 3 different groups. The primary outcome was the difference in NPVs of MRSA PCR nasal swab testing between the Prior group and the other groups. Confidence intervals for these differences were calculated using the standard error derived with the delta method. The NPV of MRSA PCR nasal swab testing for CAP has been reported to be around 98–99% [21, 27], and it was felt that the NPV should remain above 96%–97% for the test to still be useful, therefore a difference of 2% was selected as the noninferiority margin. This narrow margin was intended to increase the certainty in determining if a testing strategy was truly noninferior to testing prior to mupirocin and avoid the possibility that a high NPV was solely due to a low frequency of MRSA infections. This choice of noninferiority margin is also consistent with the finding that an approximately 5% or less antibiotic failure rate is what would be acceptable to >80% of infectious disease consultants when treating CAP [35]. Statistical analyses were performed using GraphPad Prism version 10.4.0 unless otherwise stated.

## RESULTS

A total of 1969 patients were assessed for eligibility for this study, 935 (47.5%) of whom were excluded primarily because they did not receive mupirocin at any point during their hospitalization or did not have sufficient cultures (Figure 1). Of the remaining 1034 patients, 508 (49.1%) had a MRSA PCR nasal swab collected prior to the start of mupirocin decolonization (Prior group). Among the 526 (50.9%) patients who had MRSA PCR nasal swabs collected after the start of mupirocin decolonization, 388 (73.8%) of those patients had nasal swabs collected within 7 days after the start of mupirocin decolonization (0–7 day group) and 138 (26.2%) had nasal swabs collected >7 days after the start of mupirocin decolonization (>7 day group).

Baseline characteristics are shown in Table 1. Median age and sex were similar between all 3 groups with a male predominance in all groups. Hospital length of stay, ICU length of stay, and time from admission to MRSA PCR swab collection were significantly different between the 3 groups, with patients in the >7 day group having the longest durations of all 3 measures. Although there were significant differences between the 3 groups in the time from admission to the receipt of the first dose of mupirocin, the median time for all 3 groups was <24 hours.

**Table 1.** Baseline Characteristics

	No. (%)			
	MRSA PCR Nasal Swab Testing Collected:			
Characteristic	Prior to the Start of Mupirocin (n = 508)	>0 to ≤7 d After the Start of Mupirocin (n = 388)	>7 d After the Start of Mupirocin (n = 138)	*P* Value^e^
Age, median (IQR), y	63 (51–73)	63 (51–72)	59 (45–69.25)	.372
Sex				.801
Female	218 (43)	172 (44)	57 (41)	
Male	290 (57)	216 (56)	81 (59)	
Length of stay, median (IQR), d	10.3 (5.5–19.1)	9.0 (5.2–17.1)	32.4 (22–48.5)	<.001
ICU Length of stay, median (IQR), d	3.2 (1.6–6.7)	2.5 (1.1–5.7)	10.7 (4.5–20.1)	<.001
Time from admission to MRSA PCR nasal swab collection, median (IQR), h	9.4 (5.3–24.1)	32.4 (13.2–80.1)	363.5 (252.1–586.7)	<.001
Time from admission to first dose of mupirocin, median (IQR), h	23.8 (10.3–114.2)	8.3 (5.4–13.8)	14.2 (6.5–41.3)	<.001
Total number of doses of mupirocin received, median (IQR)	9 (7–10)	9.5 (8–10)	10 (9–12)	<.001
Total number of MRSA PCR nasal swabs collected, median (range)	1 (1–3)	1 (1–2)	1 (1–2)	.216
MRSA PCR nasal swab collected while in ICU	346 (68)	252 (65)	56 (41)	<.001
Number of relevant cultures collected, median (IQR)^a^	3 (2–5)	3 (2–5)	3 (2–5)	.308
Number of patients with relevant cultures of the following types				
Respiratory cultures^b^	227 (45)	184 (47)	96 (70)	<.001
Blood cultures^b^	478 (94)	342 (88)	114 (83)	<.001
Skin, soft tissue, or bone cultures^b^	53 (10)	27 (7)	7 (5)	.056
Urinary cultures^b^	204 (40)	168 (43)	52 (38)	.444
Intra-abdominal cultures^b^	28 (6)	26 (7)	8 (6)	.755
Other cultures^b,c^	29 (6)	17 (4)	5 (4)	.495
Suspicion for pneumonia^d^	214 (42)	166 (43)	91 (66)	<.001
Mechanical ventilation prior to collection of MRSA PCR nasal swabs	117 (23)	128 (33)	93 (66)	<.001
*Staphylococcus aureus* detected via PCR testing of nasal swab	159 (31)	114 (29)	21 (15)	.001
MRSA detected via PCR testing of nasal swab	77 (15)	51 (13)	7 (5)	.008
Incidence of MRSA infection	27 (5)	19 (5)	2 (1)	.077

Abbreviations: ICU, intensive care unit; IQR, interquartile range; MRSA, methicillin-resistant Staphylococcus aureus; PCR, polymerase chain reaction.

^a^Relevant cultures were defined as a bacterial culture collected up to 3 d before or 7 d after MRSA PCR nasal swab collection for which a result was available in the EHR.

^b^Numbers refer to the number of patients with at least 1 relevant culture of the specified culture type.

^c^Other cultures include any standard bacterial cultures from sites not listed above. Specialized or selective bacterial cultures that are not routinely used to culture *Staphylococcus aureus* were excluded.

^d^Suspicion for pneumonia was defined as having a relevant respiratory culture collected, radiographic findings suggestive of pneumonia, and clinical concern for pneumonia. The criteria used to determine this are further defined in the methods section.

^e^Kruskal–Wallis test for continuous variables; χ^2^ test for categorical variables.

**Figure 2.**
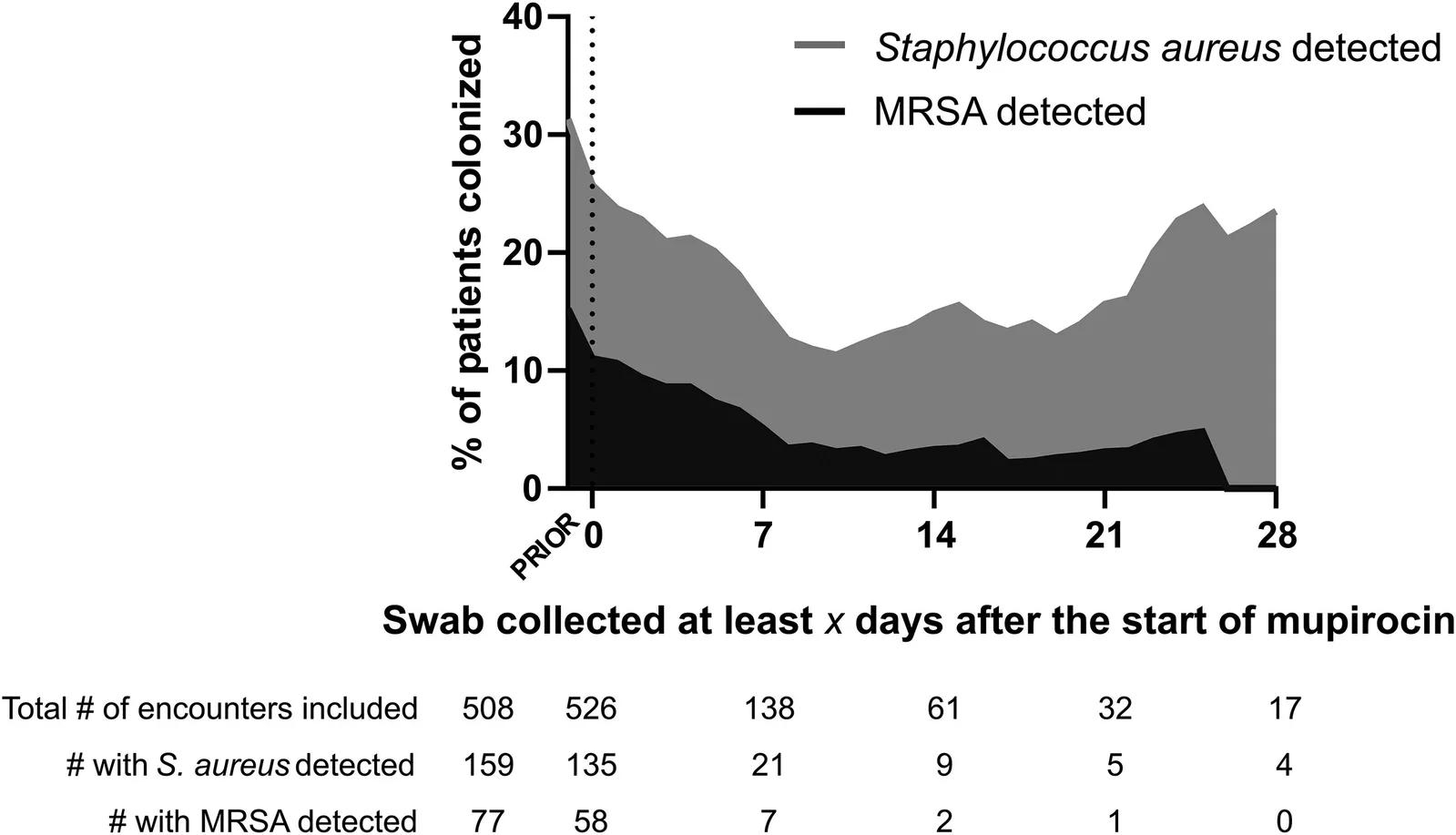
Colonization rates based on time since the start of mupirocin. Abbreviation: MRSA, methicilin-resistant *Staphylococcus aureus*.

The number and types of cultures collected, the rates of mechanical ventilation prior to collection of nasal swabs, and the percentage of patients with suspicion for pneumonia were overall similar between the patients in the Prior group and the 0–7 day group but differed significantly from the >7 day group. Likewise, rates of *S. aureus* colonization, MRSA colonization, and MRSA infection were very similar between the Prior and 0–7 day groups, but all 3 rates were lower in the >7 day group. Rates of detection of MRSA colonization were 15% in the Prior group, 13% in the 0–7 day group and 5% in the >7 day group. Grouping MRSA colonization rates based on the minimum number of days after the start of mupirocin before swab collection (Figure 2) revealed a decrease in MRSA colonization rates when swabs were collected at least 1 week after the start of mupirocin. This decrease persisted when restricting the analysis to swabs collected even further from the time of mupirocin (Figure 2). The incidence of MRSA infection in the >7 day group was 1% versus 5% in the other 2 groups.

As shown in Table 2, performance of the MRSA PCR nasal swabs was similar between the Prior group and the 0–7 day group with regards to sensitivity (81.5% vs 84.2%), specificity (88.6% vs 90.5%), PPV (28.6% vs 31.4%), and NPV (98.8% vs 99.1%). The >7 day group had comparable specificity (94.9%) and NPV (98.5%), but this was driven by the low incidence of MRSA infections in this group as there were only 2 MRSA infections, neither of which was correctly identified by MRSA PCR testing, hence a calculated sensitivity and PPV of 0%.

**Table 2.** Performance of MRSA PCR Nasal Swab Testing Relative to Timing of Initiation of Mupirocin

	No. (%)		
	MRSA PCR Nasal Swab Testing Collected:		
Test Characteristic	Prior to the Start of Mupirocin (n = 508)	>0 to ≤7 d After the Start of Mupirocin (n = 388)	>7 d After the Start of Mupirocin (n = 138)
False negative	5 (1)	3 (1)	2 (1)
True positive	22 (4)	16 (4)	0 (0)
False positive	55 (11)	35 (9)	7 (5)
True negative	426 (84)	334 (86)	129 (93)
Sensitivity (95% CI)	81.5% (63.3–91.8)	84.2% (62.4–94.5)	0.0% (0.0–82.2)
Specificity (95% CI)	88.6% (85.4–91.1)	90.5% (87.1–93.1)	94.9% (89.8–97.5)
PPV (95% CI)	28.6% (19.7–39.5)	31.4% (20.3–45.0)	0.0% (0.0–35.4)
NPV (95% CI)	98.8% (97.3–99.5)	99.1% (97.4–99.8)	98.5% (94.6–99.7)
Positive Likelihood Ratio (LR+)	7.13	8.88	0
Negative Likelihood Ratio (LR-)	0.21	0.17	1.05

Abbreviations: CI, confidence interval; NPV, negative predictive value.

**Figure 3.**
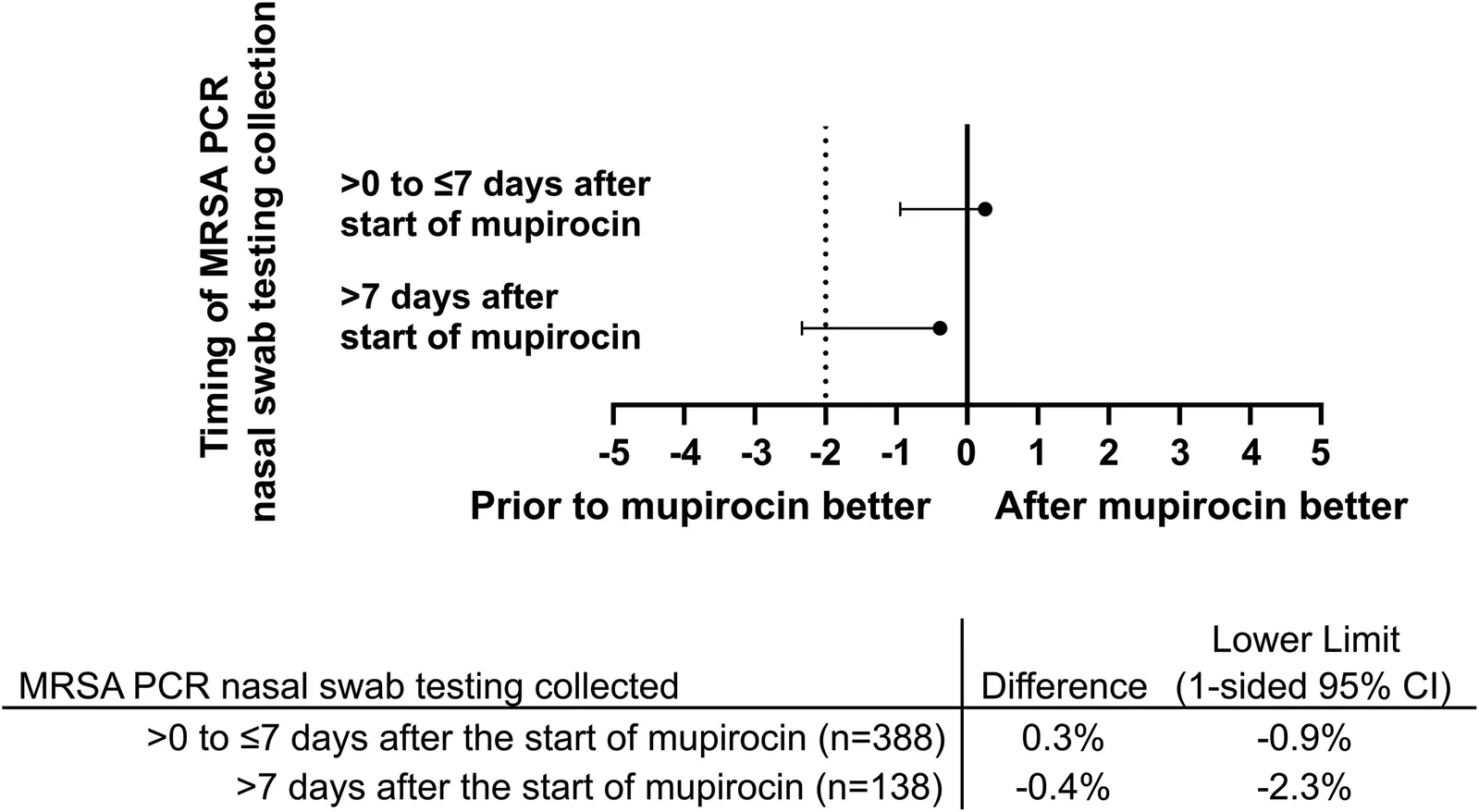
Differences in the negative predictive value (NPV) of MRSA PCR testing based on timing of swab collection relative to mupirocin decolonization. Abbreviations: MRSA, methicillin-resistant *Staphylococcus aureus*; PCR, polymerase chain reaction

To directly compare the performance of MRSA PCR nasal swab testing based on timing of swab collection relative to mupirocin decolonization, we analyzed the difference between the NPV of MRSA PCR testing in the 0–7 day group and >7 day group using a noninferiority margin of 2% (Figure 3). The 0–7 day group had an increase of 0.3% in the NPV, when compared with the Prior group as a reference, with a 1-sided 95% confidence interval lower bound of −0.9%, thus supporting the hypothesis that swab testing within 1 week of the start of mupirocin decolonization is noninferior to testing prior to the start of mupirocin. The >7 day group had a decrease in the NPV of −0.4% with a 1-sided 95% confidence interval lower bound of −2.3%, so noninferiority could not be established for swabs collected >7 days after the start of mupirocin.

Patients in all 3 groups had a median of 3 relevant cultures collected (Table 1). The most common culture types were blood cultures (934/1034 encounters, 90%), followed by respiratory cultures (507/1034 encounters; 49%) and urinary cultures (424/1034 encounters; 41%). MRSA PCR nasal swab testing performance by culture type is shown in Table 3. Among all 3 groups of patients, the NPV of the MRSA PCR test remained >96% for blood, respiratory, urinary, and skin, soft tissue, or bone cultures regardless of timing relative to initiation of mupirocin decolonization.

**Table 3.** Performance of MRSA PCR Nasal Swab Testing Based on Culture Type

Culture Type	D Relative to Mupirocin Initiation		Encounters With Indicated Culture Type	Incidence of MRSA (n)	FN	TP	FP	TN	Sensitivity (95% CI)	Specificity (95% CI)	PPV (95% CI)	NPV (95% CI)	Difference in NPV (Lower Bound of 1-Sided 95% CI)
Blood			934	1.7% (16)	3	13	113	805					
	Prior		478	1.9% (9)	2	7	66	403	77.8% (45.3–96.1)	85.9% (82.5–88.8)	9.6% (4.7–18.5)	99.5% (98.2–99.9)	N/A
	>0 to ≤7 d		342	2.0% (7)	1	6	41	294	85.7% (48.7–99.3)	87.8% (83.8–90.9)	12.8% (6.0–25.2)	99.7% (98.1–100.0)	.2% (−.6%)
	>7 d		114	0.0% (0)	0	0	6	108	N/A	94.7% (89.0–97.6)	.0% (.0–39.0)	100.0% (96.6–100.0)	.5% (−.1%)
Respiratory			507	4.3% (22)	6	16	46	439					
	Prior		227	6.6% (15)	3	12	23	189	80.0% (54.8–93.0)	89.2% (84.3–92.7)	34.3% (20.8–50.9)	98.4% (95.5–99.6)	N/A
	>0 to ≤7 d		184	2.7% (5)	1	4	17	162	80.0% (37.6–99.0)	90.5% (85.3–94.0)	19.1% (7.7–40.0)	99.4% (96.6–100.0)	1.0% (−.8%)
	>7 d		96	2.1% (2)	2	0	6	88	.0% (.0–82.2)	93.6% (86.8–97.0)	.0% (.0–39.0)	97.8% (92.3–99.6)	−.7% (−3.6%)
Urinary			424	0.7% (3)	1	2	63	358					
	Prior		204	0.5% (1)	1	0	32	171	.0% (.0–89.5)	84.2% (79.6–88.0)	.0% (.0–78.0)	99.4% (97.4–100)	N/A
	>0 to ≤7 d		168	1.2% (2)	0	2	27	139	100.0% (26.6–100)	83.7% (78.5–87.9)	6.9% (1.8–18.8)	100.0% (98.1–100)	.6% (−.4%)
	>7 d		52	0.0% (0)	0	0	4	48	N/A	92.3% (83.9–96.5)	.0% (.0–40.4)	100.0% (94.7–100)	.6% (−.4%)
Skin, soft tissue, or bone			87	17.2% (15)	1	14	4	68					
	Prior		53	17.0% (9)	1	8	4	40	88.9% (56.5–99.4)	90.9% (78.8–96.4)	66.7% (39.1–86.2)	97.6% (87.4–99.9)	N/A
	>0 to ≤7 d		27	22.2% (6)	0	6	0	21	100.0% (61.0–100)	100.0% (84.5–100)	100.0% (61.0–100)	100.0% (84.5–100)	2.4% (−1.5%)
	>7 d^a^		7	0.0% (0)	0	0	0	7	N/A	100.0% (64.8–100)	N/A	100.0% (64.6–100)	− (−)
Intra-abdominal			61	1.6% (1)	1	0	3	58					
	Prior		28	0.0% (0)	0	0	1	27	N/A	96.4% (82.3–99.8)	.0% (0.0–94.9)	100.0% (87.5–100)	N/A
	>0 to ≤7 d		26	3.8% (1)	1	0	2	23	.0% (.0–94.9)	92.0% (75.0–98.6)	.0% (.0–82.2)	95.8% (79.8–99.8)	−4.2% (−10.9%)
	>7 d^a^		8	0.0% (0)	0	0	0	8	N/A	100.0% (67.6–100)	N/A	100.0% (67.6–100)	− (−)
Other			51	2.0% (1)	1	0	6	44					
	Prior		29	0.0% (0)	0	0	3	26	N/A	89.7% (73.6–96.4)	.0% (.0–56.2)	100.0% (87.1–100)	N/A
	>0 to ≤7 d		17	5.9% (1)	1	0	2	14	0.0% (.0–94.9)	87.5% (64.0–97.8)	.0% (.0–82.2)	93.3% (70.2–99.7)	−6.7% (−17.3%)
	>7 d^a^		5	0.0% (0)	0	0	1	4	N/A	80.0% (37.6–99.0)	.0% (.0–94.9)	100.0% (51.0–100)	− (−)

Abbreviations: CI, confidence interval; MRSA, methicillin-resistant *Staphylococcus aureus*; NPV, negative predictive value.

^a^Difference in NPV and lower bound of 1-sided 95% CI not calculated due to limited data.

A sub analysis of patients with suspicion for pneumonia identified 214 patients (42.1% of the initial group) in the Prior group, 166 pts (42.8%) in the 0–7 group and 91 (65.9%) in the >7 day group. Similar to the primary analysis, patients with suspicion for pneumonia in the >7 day group had a longer length of hospital and ICU stay, and were more likely to have been mechanically ventilated prior to collection of MRSA PCR nasal swabs compared with the Prior and 0–7 day groups (Supplementary Table 1). MRSA colonization and incidence of MRSA infection also mirrored the baseline characteristics of all patients, with similar rates in the Prior and 0–7 day groups in the pneumonia cohort and lower rates of both in the >7 day groups. In the cohort with suspicion for pneumonia, performance of MRSA PCR nasal swabs was again similar between the Prior and 0–7 day group with regards to sensitivity (73.3% vs 71.4%), specificity (91.0% vs 89.9%), and NPV (97.8% vs 98.6%) (Table 4). Specificity and NPV also remained high in >7 day group at 94.9% and 98.5%, respectively.

**Table 4.** Performance of MRSA PCR Nasal Swab Testing in Patients With Suspicion for Pneumonia

	No. (%)		
	MRSA PCR Nasal Swab Testing Collected:		
Test Characteristic	Prior to the Start of Mupirocin (n = 214)	>0 to ≤7 d After the Start of Mupirocin (n = 166)	>7 d After the Start of Mupirocin (n = 91)
False negative	4 (2)	2 (1)	2 (2)
True positive	11 (5)	5 (3)	0 (0)
False positive	18 (8)	16 (10)	5 (5)
True negative	181 (85)	143 (86)	84 (92)
Sensitivity (95% CI)	73.3% (48.1–89.1)	71.4% (35.9–94.9)	0.0% (0.0–82.2)
Specificity (95% CI)	91.0% (86.2–94.2)	89.9% (84.3–93.7)	94.4% (87.5–97.6)
PPV (95% CI)	37.9% (22.7–56.0)	23.8% (10.6–45.1)	0.0% (0.0–43.5)
NPV (95% CI)	97.8% (94.6–99.2)	98.6% (95.1–99.8)	97.7% (91.9–99.6)
Positive Likelihood Ratio (LR+)	8.11	7.10	0.00
Negative Likelihood Ratio (LR-)	0.29	0.32	1.06

Abbreviations: MRSA, methicillin-resistant *Staphylococcus aureus*; NPV, negative predictive value; PCR, polymerase chain reaction.

## DISCUSSION

Despite well-founded concerns about the ability to use MRSA PCR nasal swab testing in patients who have begun mupirocin decolonization, results of this study suggest that mupirocin does not necessarily preclude the use of MRSA PCR nasal swab testing as an antimicrobial stewardship tool. MRSA PCR testing, when performed within 7 days of the initiation of mupirocin decolonization, was found to be noninferior to testing prior to decolonization (Figure 3).

MRSA PCR nasal swab testing performance was consistent across various culture types with high NPVs. Given the large numbers of blood, respiratory, and urine cultures included in this study, there is strong confidence in the performance of swab testing for these culture types. Furthermore, MRSA PCR nasal swab testing performance was also preserved among the subgroup of patients with suspicion for pneumonia (Table 4). Even within this smaller subgroup, noninferiority was demonstrated despite the strict 2% noninferiority margin (Supplementary Figure 1).

It is possible that the results of this study are due to high rates of mupirocin resistance among the hospitals included in this study. Mupirocin resistance is not routinely tested in our hospital system, however global prevalence of mupirocin resistance in MRSA is approximately 14% [36]. However, resistance is unlikely to explain the results in this study as MRSA colonization rates were substantially lower in the >7 day group (Table 1) and decreased the further from the start of mupirocin that swabs were collected (Figure 2). Furthermore, when the subgroup of patients who received mupirocin within the first 24 hours of hospitalization were compared with patients who were excluded from the primary analysis because they did not receive mupirocin, the patients who never received mupirocin were noted to have stable to increased MRSA colonization rates the farther from admission that they were tested (Supplementary Figure 2). This not only supports the well-established practice of mupirocin decolonization for reducing MRSA colonization but also suggests that the NPV remains high after the start of mupirocin because, in part, the incidence of MRSA is lower. Furthermore, this likely explains the results observed in the >7 day group, where the NPV remained high despite the test having a sensitivity of 0% and missing the only true MRSA infections. These results suggest that while the NPV may still be high, there is little utility in routinely using a MRSA PCR nasal swab test more than 1 week after mupirocin decolonization. This finding may be a useful part of stewardship protocol development to reduce unnecessary testing.

The reason MRSA PCR testing maintains its performance after the start of mupirocin decolonization is likely in large part because it detects DNA, which does not require the presence of viable organisms. This is consistent with the findings that MRSA PCR testing is more likely to be positive than culture-based methods after patients have begun antibiotics [37]. In support of this explanation, despite finding a time-dependent correlation with decreased MRSA PCR positivity (Figure 2) there was only a weak correlation between the number of doses of mupirocin received and the PCR positivity rate (Supplementary Figure 3), again suggesting that the time to clear DNA is the more important factor affecting the duration in which a PCR remains positive.

### Limitations

This study has several limitations. Firstly, the study was conducted at a single organization (Vanderbilt Health System) within 1 region, which may limit generalizability. However, 8 ICUs, representing several different specialties, across 4 different hospitals were included. Secondly, this study was limited to patients who spent at least some time in the ICU during their admission, which may limit generalizability to lower-acuity patients. Thirdly, its retrospective design resulted in significant differences in the study groups (MRSA swab prior to, >0 to ≤7 days and >7 days after start of decolonization) that may impact the NPV of the MRSA PCR nasal swab (for example, the latter group had longer median hospital and ICU length of stay, higher rates of mechanical ventilation prior to swabs and longer median time from admission to first dose of mupirocin). However, the findings in the subgroup specifically identified as having suspicion for pneumonia were similar to the overall findings, thus increasing the relevance of these results to the population for which MRSA PCR testing is likely to be used for antimicrobial stewardship decision-making.

## CONCLUSION

Determination of MRSA colonization status by nasal PCR testing remains a reliable tool to predict the likelihood of MRSA infection in patients who have begun mupirocin decolonization in the last 7 days, with high NPVs across multiple culture types. A large sample size across several hospitals, including an academic tertiary care center, as well as large number of culture types included in this study, strengthen this finding. Given the effectiveness of mupirocin decolonization, the use of MRSA PCR testing more than a week after the start of mupirocin decolonization likely has little clinical utility. These findings should be considered when designing and implementing policies regarding the concurrent use of mupirocin decolonization and MRSA PCR nasal swab testing to support the goals of infection prevention and antimicrobial stewardship programs alike.

## Supplementary Material

ciag183_Supplementary_Data

## Data Availability

The data underlying this article will be shared on reasonable request to the corresponding author.

## Supplementary Data

Supplementary materials are available at *Clinical Infectious Diseases* online. Consisting of data provided by the authors to benefit the reader, the posted materials are not copyedited and are the sole responsibility of the authors, so questions or comments should be addressed to the corresponding author.
