# Supplementary material for: Evaluation of the Impact of Universal Mupirocin Decolonization in Intensive Care Units on the Utility of Methicillin-Resistant Staphylococcus aureus Polymerase Chain Reaction Nasal Swab Testing
Source: Clin Infect Dis. 2026 Mar 16;83(2):e240–8. doi: 10.1093/cid/ciag183 (PMC13537128; doi:10.1093/cid/ciag183)
Supplement: Supplementary file 1 [file ciag183_supplementary_data.docx]

**SUPPLEMENTARY DATA**

**Figure S1.** Differences in the Negative Predictive Value (NPV) of MRSA PCR Testing Based on Timing of Swab Collection Relative to Mupirocin Decolonization in Patients with Suspicion of Pneumonia

**Figure S2.** Colonization rates based on time since admission. (A) Mupirocin started within 24 hours of admission. (B) No mupirocin received during admission.

**Figure S3.** Colonization rates based on number of doses of mupirocin received prior to testing

**Table S1.** Baseline characteristics in patients with suspicion for pneumonia

|  |  | **No. (%)** |  |  |  |
| --- | --- | --- | --- | --- | --- |
|  |  | **MRSA PCR nasal swab testing collected:** | | |  |
| **Characteristic** | | **Prior to the start of mupirocin  (n=214)** | **>0 to ≤7 days after the start of mupirocin (n=166)** | **>7 days after the start of mupirocin (n=91)** | ***P* value** |
| Age, median (IQR), y | | 64 (50-72) | 61 (51-72) | 59 (46-69) | 0.1934 |
| Sex | |  |  |  | 0.7368 |
|  | Female | 81 (38) | 69 (42) | 34 (37) |  |
|  | Male | 133 (62) | 97 (58) | 57 (63) |  |
| Length of stay, median (IQR), d | | 12.1 (6.1-20.3) | 10.7 (6.5-18.3) | 31.4 (21.9-45.8) | <0.0001 |
| ICU Length of stay, median (IQR), d | | 4.3 (2.1-10.3) | 4.0 (1.6-8.1) | 12.7 (5.7-25.0) | <0.0001 |
| Time from admission to MRSA PCR nasal swab collection, median (IQR), h | | 9.6 (5.2-20.6) | 34.1 (13.8-99.9) | 339.0 (235.1-550.8) | <0.0001 |
| Time from admission to first dose of mupirocin, median (IQR), h | | 30.0 (10.2-125.1) | 8.3 (5.0-15.9) | 12.5 (6.1-39.8) | <0.0001 |
| Total number of doses of mupirocin received, median (IQR) | | 9 (7-10) | 10 (8-10) | 10 (9-11) | 0.0044 |
| Total number of MRSA PCR nasal swabs collected, median (range) | | 1 (1-3) | 1 (1-2) | 1 (1-2) | 0.5207 |
| MRSA PCR nasal swab collected while in ICU | | 149 (70) | 127 (77) | 44 (48) | <0.0001 |
| Number of relevant cultures collected, median (IQR)^a^ | | 4 (2.75-6) | 4 (3-5) | 4 (3-6) | 0.7741 |
| Mechanical ventilation prior to collection of MRSA PCR nasal swabs | | 72 (34) | 82 (49) | 69 (76) | <0.0001 |
| *Staphylococcus aureus* detected via PCR testing of nasal swab | | 59 (28) | 49 (30) | 14 (15) | 0.0009 |
| MRSA detected via PCR testing of nasal swab | | 29 (14) | 21 (13) | 5 (5) | 0.0077 |
| Incidence of MRSA infection | | 15 (7) | 7 (4) | 2 (2) | 0.1961 |
| ^a^relevant cultures were defined as a bacterial culture collected up to 3 days before or 7 days after MRSA PCR nasal swab collection for which a result was available in the EHR. | | | | | |
